# Th1- and Th2-related chemokine and chemokine receptor expression on the ocular surface in endotoxin-induced uveitis

**Published:** 2008-12-19

**Authors:** Liem Trinh, Françoise Brignole-Baudouin, Aude Pauly, Hong Liang, Marianne Houssier, Christophe Baudouin

**Affiliations:** 1 Department of Ophthalmology III, Quinze-Vingts National Ophthalmology Hospital, Paris, France; 2 INSERM UMR S 872, Cordeliers Biomedical Institute, Pierre et Marie Curie University, Paris Descartes University, Paris, France; 3 INSERM UMR S 592, Vision Institute, Pierre et Marie Curie University, Paris Descartes University, Paris, France; 4 Department of Toxicology, Faculty of Biological and Pharmacological Sciences, University of Paris, Paris, France

## Abstract

**Purpose:**

To determine whether the ocular surface inflammation in uveitis mimics or counteracts intraocular inflammatory pathways by directly comparing T-helper (Th) lymphocytes Th1 and Th2 markers in conjunctival and ciliary body expression in endotoxin-induced uveitis (EIU). This study used the following inflammatory markers: chemokine receptor, CC chemokine receptor 4 (CCR4), and its ligand, macrophage-derived chemokine (MDC), to evaluate Th2 participation; chemokine receptor, CCR5, to evaluate the Th1 system; and its ligand, regulated on activation normal T cell expressed and secreted (RANTES), to evaluate both Th1 and Th2 systems.

**Methods:**

Immunohistochemistry and real-time polymerase chain reaction (RT–PCR) were used to compare protein and RNA expression of CCR4, MDC, CCR5, and RANTES in the conjunctiva and ciliary body in EIU 6 h and 24 h after the lipopolysaccharide (LPS) injection and in control (without injection) Lewis rats.

**Results:**

Immunohistochemistry with CCR5, RANTES, and MDC showed an increase in fluorescent staining in the conjunctiva and ciliary body in the rats with uveitis compared to the control rats. For CCR4, immunostaining was comparable in the conjunctiva and ciliary body and did not show any clear differences between control rats and rats with EIU. For *RANTES*, *MDC*, and *CCR5*, RT-PCR showed a significantly higher RNA expression in conjunctiva and in ciliary body at 6 h compared to 24 h and controls. For *CCR4*, RT-PCR did not illustrate any significant differences in conjunctiva and in ciliary body between all groups of animals.

**Conclusions:**

Protein and RNA expressions of RANTES, MDC, and CCR5 were higher in EIU rats than in control rats in the conjunctiva and ciliary body whereas the CCR4 level was not modified in the conjunctiva and ciliary body of EIU rats when compared to controls. Th1 activation seemed to predominate in this model with high levels of CCR5 expression and no increased expression of CCR4, but Th2 participation with MDC was noted. The expression of RANTES, MDC, CCR4 and CCR5 in EIU was quite similar between the conjunctiva and the ciliary body, so conjunctival inflammation might reproduce the intraocular inflammation, probably generated by local extension and diffusion in this model. If the ocular surface mimics intraocular inflammatory pathways, the conjunctiva may provide a new and easier access for uveitis studies.

## Introduction

Uveitis is an ophthalmologic entity comprising several heterogeneous diseases, all characterized by intraocular inflammation starting initially in the uvea [1,2]. The different immune pathways involved in this process have not yet been thoroughly described. The participation of T-helper lymphocytes seems to predominate in uveitis as shown by analyses of intraocular fluids of patients with uveitis [3,4]. T-helper (Th) lymphocytes are classically composed of two subsets, Th1 and Th2, differentiated by the type of cytokines they secrete [5,6]. A new lineage of T cells, called Th17, which produces the cytokine, interleukin-17 (IL-17), has recently been described [7]. Th17 might be involved in the pathophysiology of experimental autoimmune uveitis (EAU), and Th1 could play a regulation role toward Th17 through its production of IL-27, which inhibits the secretion of IL-17 [8,9]. Th17 may play a central role in uveitis, but its function and interaction with Th1 remain to be clarified [10,11]. The Th1 response classically plays a central role in the immunopathological process of experimental autoimmune uveitis [2,12]. Analyses of intraocular fluids in human uveitis tend to favor Th1 involvement because they showed Th1 cytokines such as interferon-gamma. However, to date, the analyses have been unable to define a clear predominance of Th1 or Th2 responses [2-4]. Ocular inflammation primarily involves the uveal tract but can also extend to other ocular structures such as the retina or vitreous. The involvement of the conjunctiva in uveitis has not been studied as much. Little is known about inflammation occurring on the ocular surface of uveitic patients [13,14], but conjunctival injection is frequently observed in anterior uveitis [1].

Our laboratory developed new, noninvasive, and objective techniques to explore immunoinflammatory markers expressed on the ocular surface by conjunctival epithelial cells, using flow cytometry in impression cytology specimens [15] in a variety of ocular surface diseases [16]. Recently, our group has investigated the Th1 and Th2 inflammatory cascades by assessing CC chemokine receptor 4 (CCR4) and CCR5 expression in conjunctival cells in uveitis [17]. Indeed, CCR4 and CCR5 are known to be chemokine receptors related to the Th2 and Th1 systems, respectively. Their principal ligands are CC chemokine ligand 3(CCL3)/macrophage inflammatory protein 1α (MIP-1α), CCL4/MIP-1β, and CCL5/regulated on activation normal T cell expressed and secreted (RANTES), for CCR4, and CCL17/thymus and activation-regulated chemokine (TARC) and CCL22/macrophage-derived chemokine (MDC), for CCR5.MIP-1α is a chemokine that binds receptors of Th1 cell lines, TARC and MDC are related to Th2 and MIP-1β, and RANTES binds receptors of both Th1 and Th2 cell lines [18,19]. CCR4 was overexpressed in the epithelial conjunctival cells of uveitis, and its overexpression suggested involvement of the Th2 system on the ocular surface while participation of Th1 is usually considered to be favored in intraocular pathogenesis, at least in experimental autoimmune uveitis [6]. This previous study suggested that there may be striking differences in inflammatory pathways between intraocular tissues and the ocular surface. Therefore, each may react differently to immunogenic inflammation. The aim of the current study was to determine whether the ocular surface mimics or counteracts intraocular inflammatory pathways by directly comparing Th1 and Th2 markers between the conjunctiva (ocular surface component) and the ciliary body (representing intraocular structures) in an experimental model of uveitis, endotoxin-induced uveitis (EIU). This model of acute ocular inflammation has been used for some forms of human anterior uveitis [20] and the inflammatory cell infiltrate of the anterior segment is maximal at 24 h after lipopolysaccharide (LPS) injection [21]. In this study, these different inflammatory markers were used to asses Th1 or Th2 activation: chemokine receptor CCR4 and its ligand MDC, for Th2 participation; but also chemokine receptor, CCR5, for Th1 system; and at last its ligand, RANTES, for both the Th1 and Th2 systems. Immunohistochemistry and real-time polymerase chain reaction (PCR) were used to compare the protein and RNA expression of these markers in the conjunctiva and ciliary bodies in EIU 6 h and 24 h after LPS injection.

## Methods

### Animal groups and endotoxin-induced uveitis induction

Eighteen inbred, male, adult Lewis rats (8-10 weeks old; Centre d’élevage R. Janvier, Le Genest-St-Isle, France) were used. All animals were treated according to the Association for Research in Vision and Ophthalmology Resolution on the Human Use of Animals in Vision Research under the supervision of an independent health authority-accredited staff member for animal care and management.

Lipopolysaccharide (LPS) from *Escherichia coli* (Sigma Chemical, St Louis, MO) was dissolved in sterile pyrogen-free saline at 1 mg/ml. Uveitis was induced with a single 150 μg subcutaneous injection of LPS solution (1 mg/ml). Rats were divided into two different groups, one for histologic and immunohistochemical studies and the other for PCR analyses. Each group was composed of three subgroups of three rats (a total of nine rats per group). The first subgroup contained three control rats that were not injected, the second group was made up of three rats injected with LPS and sacrificed at 6 h, and the third subgroup consisted of three rats sacrificed 24 h after LPS injection. Rats were euthanized with an injection of pentobarbital (sodium pentobarbital, Ceva Santé Animale, Libourne, France) at a lethal dose.

### Histologic examination

After euthanasia, the eyes were enucleated, placed in the optimal cutting temperature (OCT) compound (Tissue-Tek, Zoeterwoude, the Netherlands), and snap frozen.  Seven micrometer frozen antero-posterior sections were prepared at the optic nerve level on gelatin-coated slides for histological analysis. Sections were fixed in 4% paraformaldehyde for 5 min at room temperature and stained with hematoxylin and eosin (HE). To study the modification of the conjunctival architecture and infiltration of inflammatory cells in EIU, conjunctival epithelium and stroma were examined under the light microscope with high power objectives (Leica Aristoplan; Leica Microsystemes SAS, Rueil-Malmaison, France).

### Immunohistochemistry

After sacrifice, the animals were enucleated, and the eyes were embedded in the OCT compound and snap frozen. Seven micrometer frozen antero-posterior sections were prepared at the optic nerve level on gelatin-coated slides for immunohistochemical analysis.

Sections were fixed in 4% paraformaldehyde for 20 min at room temperature, rinsed with phosphate buffered saline (PBS), and incubated in 1% Triton X-PBS (Sigma Chemical) for 20 min. After washing with PBS, sections were incubated at 4 °C overnight with one of the following specific primary antibodies diluted (1:50) in PBS containing 1% human serum albumin (Sigma Chemical) to saturate nonspecific fixation sites:

-Monoclonal mouse anti-rat CCR5 (Santa Cruz Biotechnology, Santa Cruz, CA);
-Polyclonal goat anti-rat CCR4 (Santa Cruz Biotechnology);
-Polyclonal goat anti-rat MDC (Santa Cruz Biotechnology);
-Polyclonal goat anti-rat RANTES (Santa Cruz Biotechnology;
-Immunoglobulin IgG1 mouse antibody for isotypic control (Serotec, Oxford, UK).

The sections were rinsed twice for 5 min with PBS after incubation with specific primary antibodies and then incubated for 1 h at room temperature with secondary antibodies, depending of the nature of the primary antibodies. The secondary antibodies used were Alexa Fluor 488 goat anti-mouse IgG (diluted 1:200; Invitrogen, Eugene, OR) and Alexa Fluor 488 rabbit anti-goat IgG (diluted 1:100; Invitrogen) in PBS containing 1% human serum albumin. After two washes with PBS, sections were counter-stained for 5 min with propidium iodide (Sigma Chemical), rinsed with PBS for 5 min, and mounted with Vectashield H1000 (Vector Laboratories, Burlingame, CA). The slides were examined with a confocal microscope (Nikon PCM 2000; Nikon France, Champigny-sur-Marne, France).

### Semi-quantitative reverse transcription and real-time polymerase chain reaction

The conjunctiva and ciliary body of the enucleated eyes of control rats and the experimental rats 6 h and 24 h after LPS injection were dissected separately to compare RNA expression of *RANTES*, *MDC*, *CCR4*, and *CCR5*. Total RNA was isolated with the RNeasy Mini Kit (Qiagen, Courtaboeuf, France). For each sample, single-stranded cDNA was synthesized from total RNA using dNTPs, random primers, and superscript reverse transcriptase (Invitrogen). RT–PCR was performed using cDNA and *RANTES*, *MDC*, *CCR4*, *CCR5*, and *GAPDH* (glyceraldehyde-3-phosphate dehydrogenase) as the endogenous control. TaqMan probes were purchased from Applied Biosystems (Foster City, CA). PCR reactions were performed for 40 cycles (denaturation at 95 °C for 15 s and elongation at 60 °C for 45 s) in a thermal cycler (model 7300 SDS; Applied Biosystems). The comparative threshold (C_t_) cycle method was used for the relative quantification of gene expression with the RT–PCR data. The C_t_s of *RANTES*, *MDC*, *CCR4*, and *CCR5* were normalized to the levels of the endogenous control *GAPDH* at each time after the LPS injection. The degree of change in each gene was calculated and compared with the control rats.

### Statistical analysis

The variance was analyzed using the Kruskal–Wallis test, and then the data were compared with the Mann–Whitney statistical test. The differences were judged significant when p<0.05.

## Results

### Histological analysis

**Figure 1:**
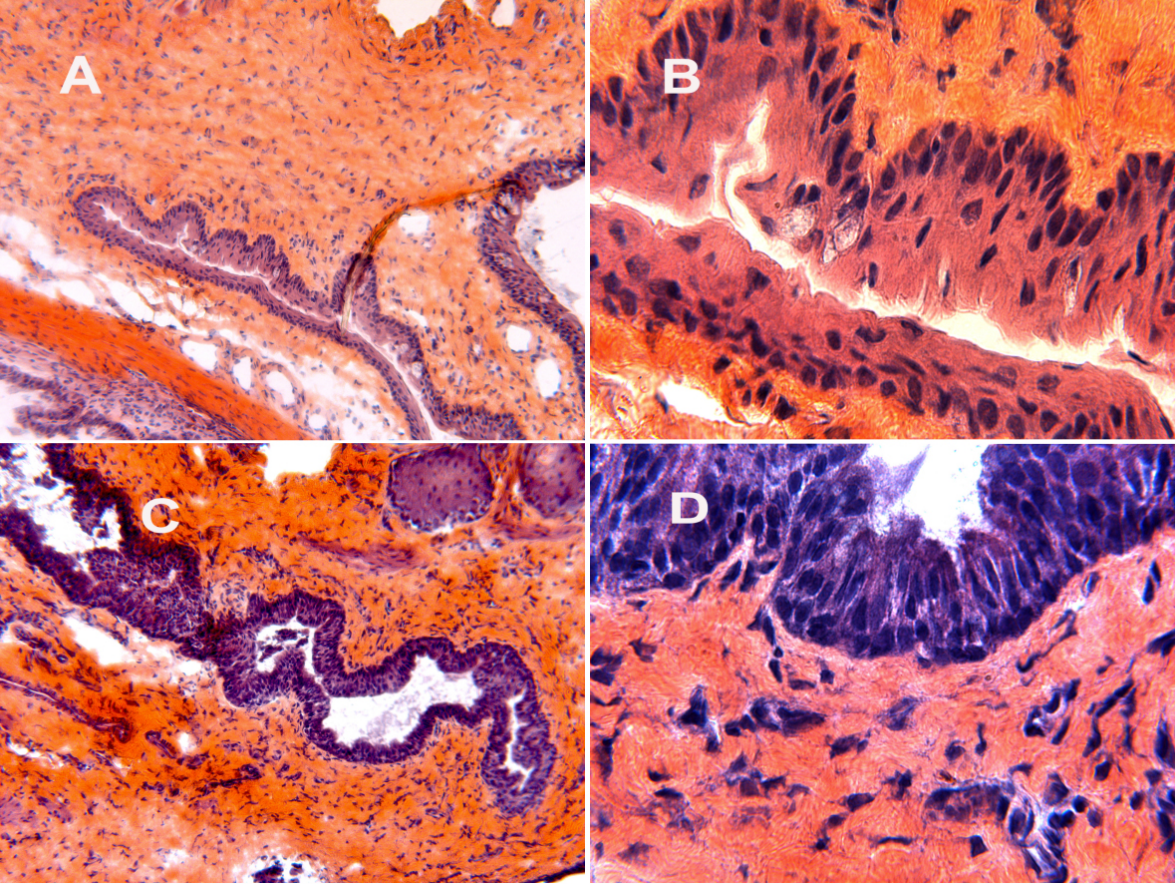
Histological micrographs. Conjunctiva in control rats with 10X (**A**) and 50X (**B**) enlargement. Conjunctiva in endotoxin-induced uveitis (EIU) 24 h after LPS injection with 10X (**C**) and a 50X (**D**) enlargement showed a disorganization of the epithelial architecture with an increase in the number of layers and the size of epithelial cells

Histological analysis of antero-posterior sections 24 h after the LPS injection showed disorganization of the epithelial architecture with an increase in the number of layers and the size of epithelial cells (Figure 1).

### Immunohistochemical analysis

**Figure 2:**
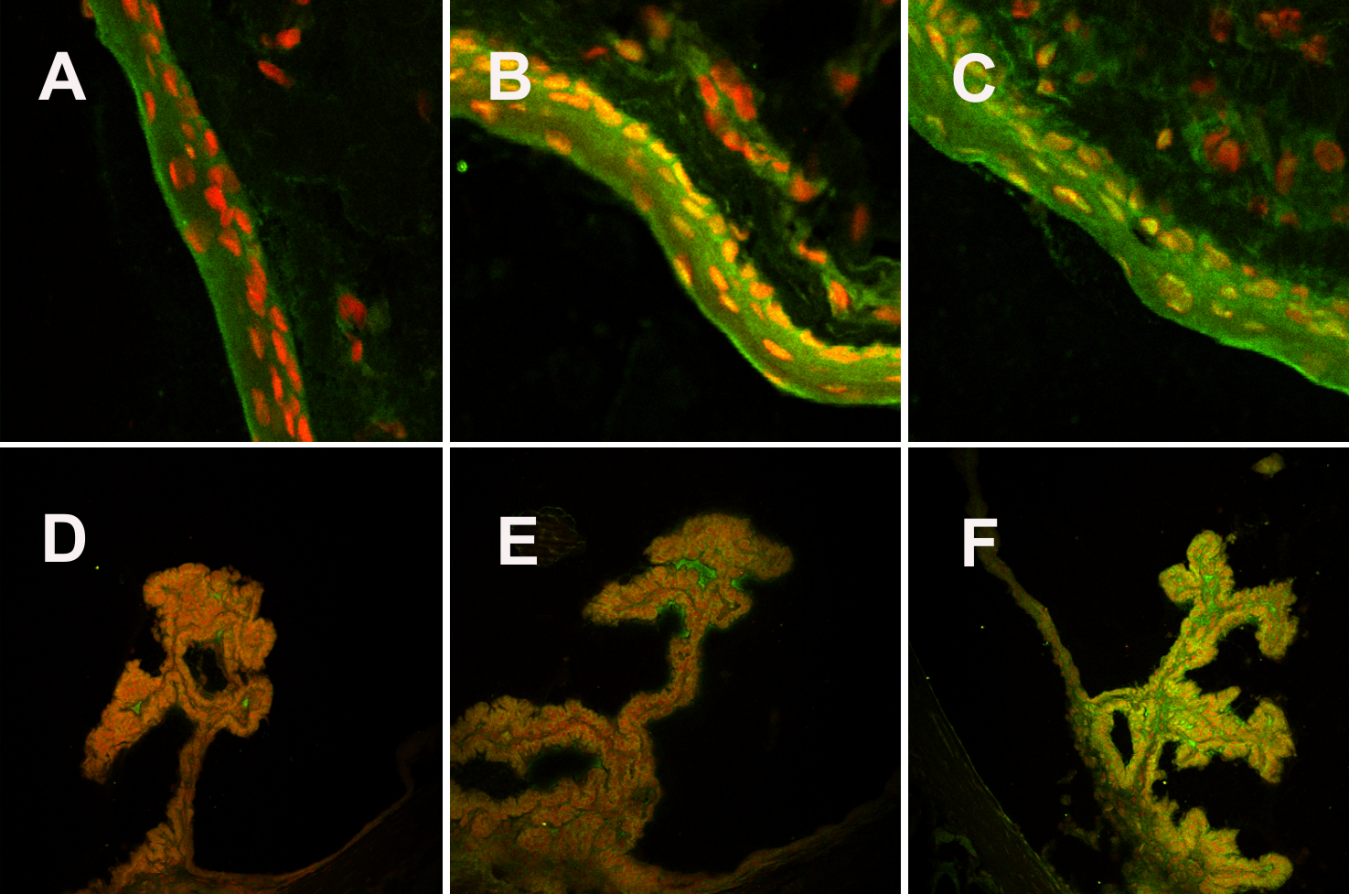
Immunostaining in a model of endotoxin-induced uveitis analyzed with a confocal microscope with 20X enlargement. Conjunctivas stained with anti-CCR5 antibody were revealed with secondary antibody, Alexa Fluor (in green), while nuclear chromatin was stained with propidium iodide (in red) in control rats (**A**) and in EIU rats 6 h (**B**) and 24 h (**C**) after LPS injection. Ciliary bodies were stained with the same antibodies in control rats (**D**) and in EIU rats 6 h (**E**) and 24 h (**F**) after LPS injection. CCR5 expression (in green) was higher in the conjunctiva and ciliary bodies of the rats with EIU than of the controls.

**Figure 3:**
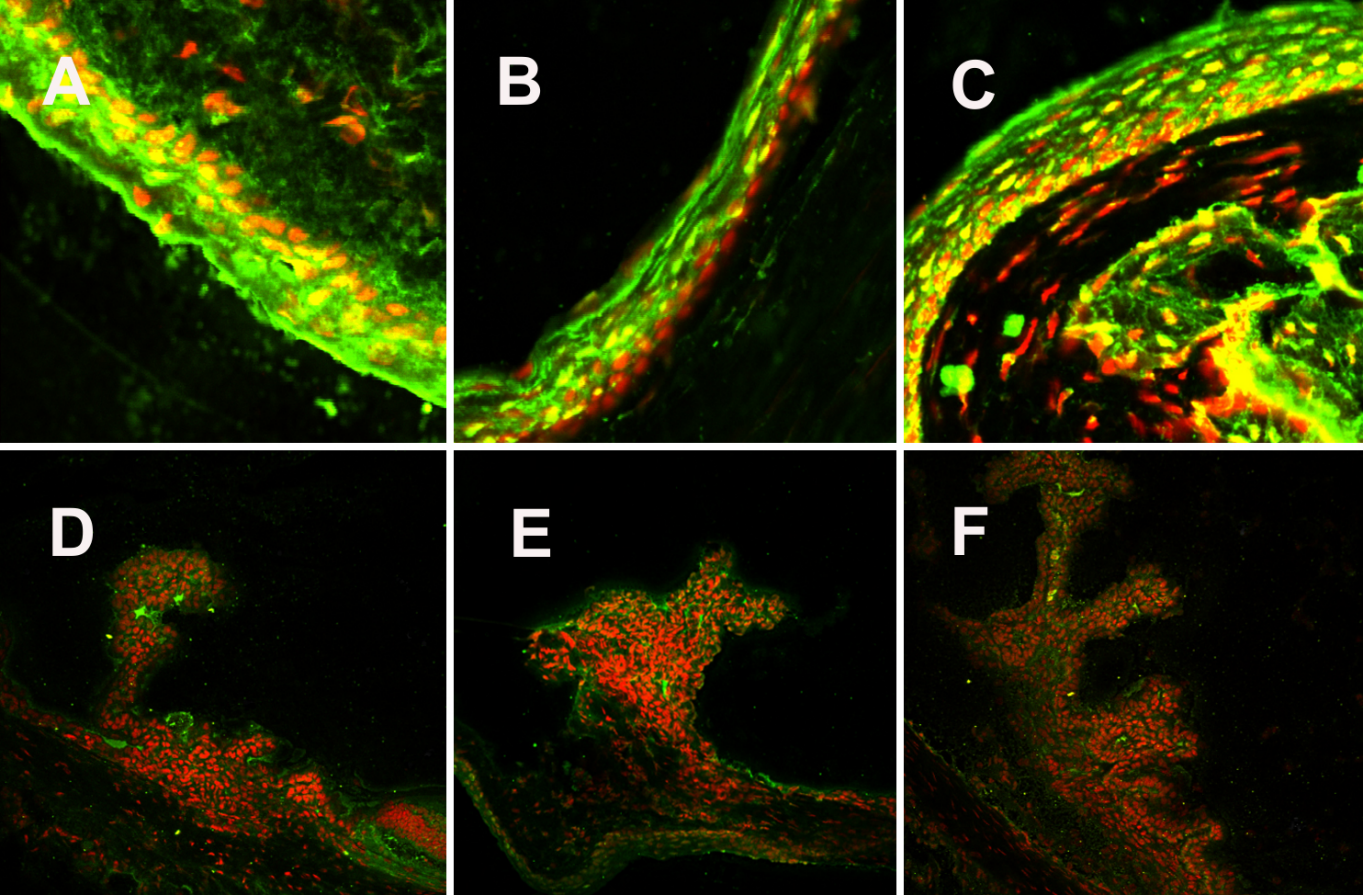
CCR4 immunostaining of conjunctiva and ciliary bodies in a model of endotoxin-induced uveitis analyzed with a confocal microscope with 20X enlargement. Conjunctiva was stained with CCR4 (in green) and propidium iodide for nuclei (in red) in control rats (**A**) and of EIU rats 6 h (**B**) and 24 h (**C**) after LPS injection. Staining of ciliary bodies was performed in control rats (**D**) and in EIU rats at 6 h (**E**) and 24 h (**F**). Immunostaining (in green) was positive in all cases but comparable in the conjunctiva and the ciliary body. Immunostaining did not show any clear differences between control rats and rats with EIU.

Immunohistochemistry with RANTES, MDC, and CCR5 showed a slight increase in fluorescent staining intensity (in green) in the conjunctival epithelium and in the ciliary body of the rats with uveitis with a peak at 24 h after the LPS injection (Figure 2) when compared to the control rats. For CCR4, immunostaining (in green) was strong but comparable in the conjunctiva and the ciliary body and did not show any clear difference between control rats and rats with EIU (Figure 3).

### Molecular biology: semi-quantitative real-time polymerase chain reaction

**Figure 4:**
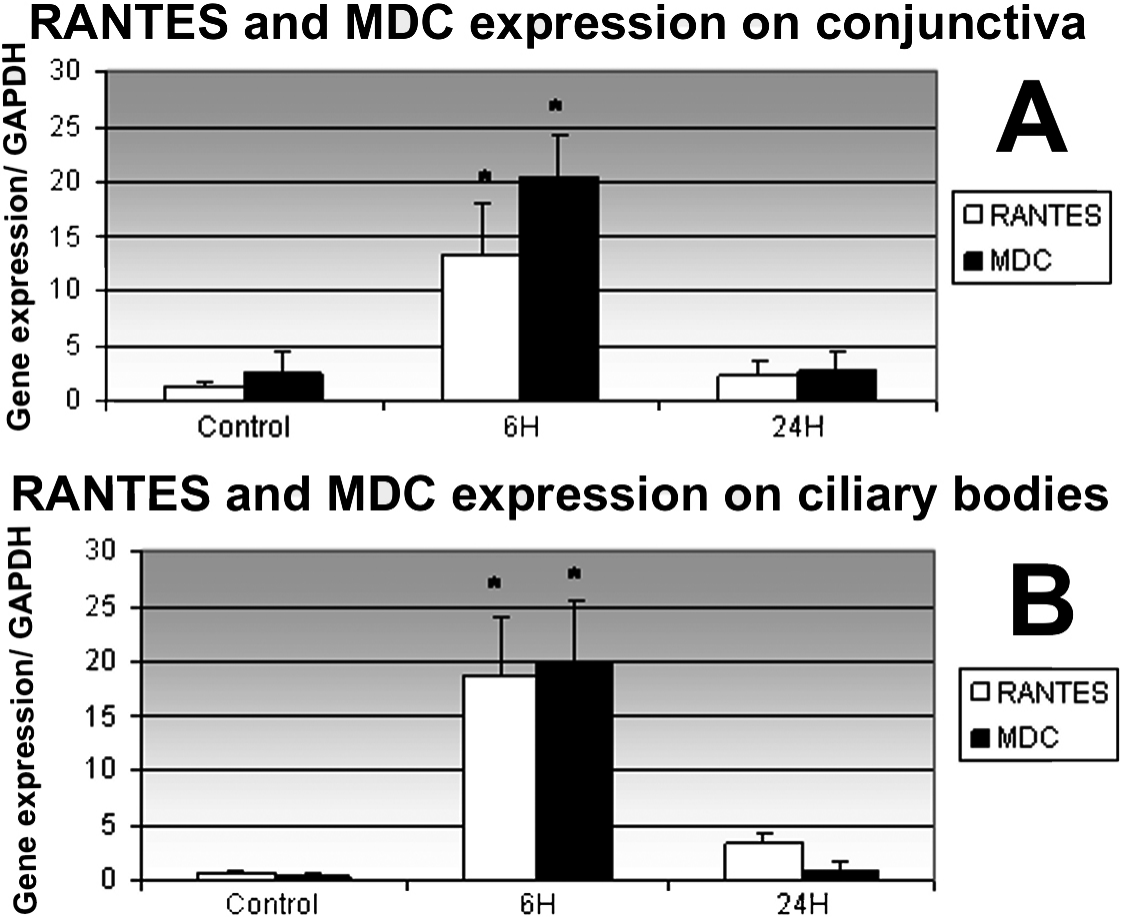
Quantification of mRNA expression of RANTES and MDC in conjunctiva and ciliary bodies by real time-PCR. mRNA expression levels of *RANTES* and *MDC* normalized to the levels of the endogenous control, *GAPDH*, were quantified in the conjunctiva (**A**) and ciliary bodies (**B**) of control rats and of rats with EIU 6 h and 24 h after LPS injection using semi-quantitative real-time PCR. Data shown are the mean±SD. An asterisk indicates that there is a significant difference between the control rats and the rats with EIU at 24 h (p<0.05).

**Figure 5:**
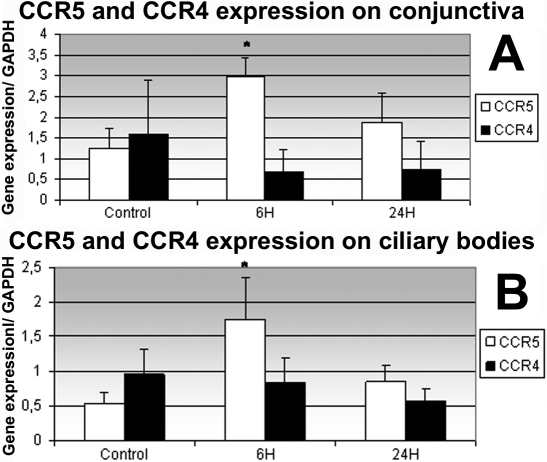
Quantification of mRNA expression of CCR5 and CCR4 in conjunctiva and ciliary bodies by real time-PCR. Semi-quantitative real-time PCR in the conjunctiva (**A**) and the ciliary body (**B**) was performed to measure the expression of *CCR5* and *CCR4* normalized to the levels of the endogenous control, *GAPDH*, in control rats and in rats with EIU 6 h and 24 h after LPS injection. Data shown are the mean±SD. An asterisk denotes that there is a significant difference between the control and EIU rats at 24 h (p<0.05).

To quantify RNA expression of *RANTES*, *MDC*, *CCR4*, and *CCR5* in the conjunctiva and ciliary body, reverse transcription polymerase chain reaction (RT–PCR) was performed on conjunctival and ciliary body extracts from controls and rats with EIU at 6 h and 24 h. RNA expression of *RANTES*, *MDC*, and *CCR5* in the conjunctiva and ciliary body was significantly higher in EIU rats at 6 h than in EIU rats at 24 h and in control rats (Figure 4 and Figure 5). For *CCR4*, there was no significant difference in the conjunctiva and in ciliary body between the controls and the EIU rats at 6 h and 24 h (Figure 5).

## Discussion

Expression of the inflammatory markers, RANTES, MDC, CCR5, and CCR4, was analyzed in the conjunctiva and ciliary bodies using two different techniques, immunostaining and molecular biology. A positive staining in immunohistochemistry was observed with all the markers studied in the conjunctiva and ciliary body of EIU rats, but this technique provided more qualitative than quantitative analyses of chemokines and chemokine receptors. To compare the level of expression of these markers, we combined the molecular biology technique with semi-quantitative real-time PCR since it is a more precise tool to quantify gene expression. In our study, semi-quantitative PCR confirmed the immunostaining results. Indeed, the protein and RNA expressions of RANTES, MDC, and CCR5 were higher in the conjunctiva and ciliary body of EIU rats (at 6 h and 24 h) than of the control rats on. In immunohistochemistry, RANTES, MDC, and CCR5 expressions peaked 24 h after the LPS injection while in PCR, their RNA expression peaked at 6 h. The time difference between the peak of protein and RNA expression could be explained by the time needed to synthesize protein from RNA. Both methods found that there were no differences in the CCR4 level between EIU rats and controls, which is quite different from our previous report in humans [17] where CCR4 was overexpressed by conjunctival epithelial cells. This difference is probably explained by the fact that EIU is a quickly systemic induced, short-term uveitis model that does not fully reproduce the complexity of human uveitis.. In this model, it is possible that in addition to the local ocular inflammation, there is an upregulation of epithelial cells by LPS systemic stimulation, which could therefore corroborate to our results published in patients with uveitis. Nevertheless, our goal was to compare surface and uvea immunopathological changes, and this model showed that expression of RANTES, MDC, CCR4, and CCR5 in EIU was quite similar in the conjunctiva and ciliary body, indicating that conjunctival inflammation might reproduce the intraocular inflammation generated in this uveitis model probably by local extension and diffusion. This study showed an overexpression of RANTES, MDC, and CCR5 in EIU animals compared to controls and confirmed previous publications in which RANTES was found by RT–PCR in the ciliary body in EIU animals [22,23]. In the literature, high levels of interleukin-1 (IL-1), IL-6, IL-10, tumor necrosis factor (TNF)-α, RANTES, interferon (IFN)-γ, monocyte chemotactic protein 1 (MCP-1), and macrophage inflammatory protein 2 (MIP-2) were found in EIU animals [21-26]. Therefore, Th1 and Th2 cytokine profiles in EIU have not been well defined, probably because inflammatory pathways in EIU mostly involve neutrophils and macrophages [27-29]. Nevertheless, lymphocytes also infiltrate the iris and ciliary body and seem to have a fundamental role in the pathogenesis of EIU [27,30,31], so discrimination of Th1/Th2 systems should be helpful in understanding lymphocyte functions in EIU. In our study, Th1 activation seemed to predominate in this model with high levels of CCR5 overexpression and no increased expression of CCR4, but Th2 participation with MDC was also noted. The Th1 response is usually thought to play a central role in other animal model studies of uveitis. Indeed, in experimental autoimmune uveitis (EAU), which is a Th1 inflammation induced by a retinal antigen, Th1 participation is recognized [2] with the overexpression of chemokines related to the Th1 system such as MIP-1 α and RANTES as well as the chemokine receptor, CCR5 [32]. In the current study, the analysis of chemokines and chemokine receptors in EIU partially confirmed the Th1 activation in the immunopathological process of EAU. There was no difference for the expression of the Th2-specific chemokine receptor, CCR4, between the EIU and control animals in our study, which is similar to findings for EAU where low levels of CCR4 were found [33].

In conclusion, chemokine and chemokine receptor analysis showed that inflammation on the ocular surface reproduced intraocular phenomena by extension in this experimental model of uveitis, EIU. Further studies on human uveitis and other experimental models of uveitis could compare the chemokine profile in intraocular fluid and ocular surface samples. If the ocular surface mimics intraocular inflammatory pathways, the conjunctiva could provide a new and easier access for uveitis studies with less invasive exams than anterior chamber paracentesis or vitrectomy samples. Chorioretinal biopsies, like conjunctival impressions, could be developed to investigate inflammatory activity and pathophysiological mechanisms in uveitis.
